# Whole genome sequencing of enriched chloroplast DNA using the Illumina GAII platform

**DOI:** 10.1186/1746-4811-6-22

**Published:** 2010-09-28

**Authors:** Robin A Atherton, Bennet J McComish, Lara D Shepherd, Lorraine A Berry, Nick W Albert, Peter J Lockhart

**Affiliations:** 1 Institute of Molecular BioSciences, Massey University, Private Bag 11 222, Palmerston North, 4442, New Zealand; 2 Allan Wilson Centre for Molecular Ecology and Evolution, Massey University, Private Bag 11 222, Palmerston North, 4442, New Zealand; 3 Massey Genome Service, Massey University, Private Bag 11 222, Palmerston North, 4442, New Zealand; 4 Institute of Fundamental Sciences, Massey University, Private Bag 11 222, Palmerston North, 4442, New Zealand

## Abstract

**Background:**

Complete chloroplast genome sequences provide a valuable source of molecular markers for studies in molecular ecology and evolution of plants. To obtain complete genome sequences, recent studies have made use of the polymerase chain reaction to amplify overlapping fragments from conserved gene loci. However, this approach is time consuming and can be more difficult to implement where gene organisation differs among plants. An alternative approach is to first isolate chloroplasts and then use the capacity of high-throughput sequencing to obtain complete genome sequences. We report our findings from studies of the latter approach, which used a simple chloroplast isolation procedure, multiply-primed rolling circle amplification of chloroplast DNA, Illumina Genome Analyzer II sequencing, and de novo assembly of paired-end sequence reads.

**Results:**

A modified rapid chloroplast isolation protocol was used to obtain plant DNA that was enriched for chloroplast DNA, but nevertheless contained nuclear and mitochondrial DNA. Multiply-primed rolling circle amplification of this mixed template produced sufficient quantities of chloroplast DNA, even when the amount of starting material was small, and improved the template quality for Illumina Genome Analyzer II (hereafter Illumina GAII) sequencing. We demonstrate, using independent samples of karaka (*Corynocarpus laevigatus*), that there is high fidelity in the sequence obtained from this template. Although less than 20% of our sequenced reads could be mapped to chloroplast genome, it was relatively easy to assemble complete chloroplast genome sequences from the mixture of nuclear, mitochondrial and chloroplast reads.

**Conclusions:**

We report successful whole genome sequencing of chloroplast DNA from karaka, obtained efficiently and with high fidelity.

## Background

Chloroplast genomes provide a wealth of information for studies in molecular ecology and evolution. Their conservative gene content and organisation have enabled researchers to isolate homologous loci for comparative studies over different evolutionary time-scales [1-7].

Obtaining the DNA sequence for chloroplast genomes can be achieved by using the polymerase chain reaction (PCR) to amplify chloroplast DNA fragments from genomic DNA (gDNA) extracts. However, this can involve up to 35 amplifications of overlapping chloroplast DNA PCR products [2,8]. While this approach is time consuming [8], it has been preferred over protocols that attempt to first separate chloroplasts from other cellular material. Reasons for this appear to be that chloroplast isolation can be troublesome in some species [9] and because rapid chloroplast isolation protocols often produce template which is still contaminated by large quantities of nuclear DNA [10]. Nevertheless, given the depth of sequencing coverage with the Illumina GAII sequencing platform, we were interested to investigate whether this alternative approach could be used for sequencing whole chloroplast genomes without the need for whole genome PCR amplification. Here we report findings which demonstrate that, even with small amounts of chloroplast DNA, and in the presence of large amounts of nuclear DNA, Illumina short read sequencing provides a practical approach for obtaining complete chloroplast genome sequences.

## Methods

### Chloroplast isolation

Fresh leaf material was obtained from two cultivated karaka trees originating in Rekohu/Chatham Islands and the Kermadec Islands, New Zealand. Leaf material was collected and processed immediately for the sample from the Chatham Islands and within 3 h for the sample from the Kermadec Islands. Leaf samples weighing 2.5-5 g were excised from living trees and processed as follows. Chloroplasts were isolated using a protocol originally designed for isolating chloroplasts from *Arabidopsis thaliana *[11] with minor modifications: (i) the leaf material was homogenised using an Ultra-Turrax homogeniser with an N18 rotor (Janke & Kunkel IKA, Hamburg, Germany); (ii) the homogenate was passed through a double layer of washed and autoclaved nappy (diaper) liner (Johnson & Johnson Ltd.) rather than through Miracloth (Calbiochem); and (iii) the final centrifugation step was carried out using a Sorvall SS32 angled rotor, rather than a swinging bucket rotor. After the final centrifugation step, DNA was extracted from pooled chloroplasts for each tree sample using a DNEasy Plant Mini Kit (Qiagen) following the manufacturer's instructions. Genomic DNA (gDNA) was extracted from silica-dried karaka leaf material from the same accessions using a DNEasy Plant Mini Kit (Qiagen).

### Multiply-primed rolling circle amplification

Multiply-primed rolling circle amplification (RCA) was used to produce an abundance of purified chloroplast DNA template in preparation for sequencing [12]. This technique involves isothermal, strand-displacing amplification using multiple primers and is capable of yielding a large amount of product from very little starting DNA template [13]. Phi29, the DNA polymerase used in multiply-primed RCA, is reported to have a very low level of amplification bias making the template suitable for whole genome sequencing [14]. Chloroplast-enriched DNA (cpDNA) from both karaka samples was amplified in this way using a REPLI-g™ Mini Kit (Qiagen) following the manufacturer's instructions, with the exception that samples were incubated at room temperature for 9 min rather than the recommended 3 min. This extension time consistently produced better results with different plant samples. The kit produced ~5 μg of product for each sample.

### Confirmation of chloroplast DNA enrichment

Genomic DNA (gDNA), chloroplast-enriched DNA (cpDNA) and RCA amplified chloroplast-enriched DNA (RCAcpDNA) from the Chatham Island sample were quantified fluorometrically using the Quant-iT™ dsDNA HS assay kit on a Qubit™ Quantitation Platform (Invitrogen). The concentration of the gDNA, cpDNA and RCAcpDNA was 110 ngμL^-1^, 20 ngμL^-1 ^and 104 ngμL^-1^, respectively, in a total volume of 50 μL of AE buffer (Qiagen). The purity of gDNA, cpDNA and RCAcpDNA samples was determined by A_260_/A_280 _and A_260_/A_230 _ratios on a NanoDrop (NanoDrop Technologies) spectrophotometer. Enrichment for chloroplast DNA was determined by quantitative real-time PCR (qPCR) with gDNA, cpDNA and RCAcpDNA templates; the quantity of the plastid gene *psbB *was determined relative to nuclear encoded 18S *rRNA *by comparative quantification [15]. Gene-specific primers were designed for *psbB *(*psbB *F 5'GGGGGTTGGAGTATCACAGG3'; *psbB *R 5'CCAAGAAGCACAAGCCAGAA3', 103 bp amplicon) using Primer3 [16] and primers for 18S are described by Zhu and Altmann [17]. qPCR was performed using Lightcycler480 SYBR Green1 Master (Roche Diagnostics) reagents in a Rotor Gene 3000 instrument (Corbett Research) with four technical replicates per sample. Template DNA was diluted 20-fold for cpDNA, and 100-fold for gDNA and RCAcpDNA samples for qPCR. The qPCR cycling conditions were: 95°C 10 min, (95°C 10 s, 60°C 15 s, 72°C 20 s) × 40 cycles with fluorescent detection at 72°C and during the final melt. Melt curve analysis confirmed the amplification of a single product.

### Illumina GAII sequencing

The RCAcpDNA samples from both accessions were sequenced by Massey Genome Service (Massey University, Palmerston North, New Zealand). A 75 bp paired-end run was performed on the Illumina GAII with the two samples described here in a single lane along with four other samples from a separate experiment. Samples were prepared for sequencing as follows: genomic DNA libraries were prepared by fragmenting purified genomic DNA using a nebulisation kit (Invitrogen), paired-end index adaptor ligation (Illumina) and 18 cycles of PCR enrichment using the Illumina Paired-End Genomic DNA library preparation kit, Illumina Multiplex Oligonucleotide library preparation kit and Illumina Multiplex Paired-End Genomic DNA library preparation protocol. The enriched libraries were quantified using an ND-1000 NanoDrop spectrophotometer (NanoDrop Technologies) and quality checked by Agilent 2100 Bioanalyzer, DNA 1000 Labchip kit assay. The libraries were then diluted to a 10 nM concentration using EB buffer (Qiagen) and quantified for optimal cluster density using the LightCycler^® ^480 system Absolute Quantification protocol (Roche Diagnostics) and the LightCycler^® ^480 SYBR Green I Master kit (Roche Diagnostics). The libraries were pooled at equal molarity and amplified in one flow cell lane on the Illumina Cluster Station instrument at a density of 140,000 clusters per tile and a molarity of 13 pM using the Illumina Paired-End Cluster Generation kit v2. The amplified libraries were sequenced on the Illumina GAII instrument using 4 Illumina 36 cycle SBS sequencing kits (v3), Illumina Multiplex Sequencing Primers and PhiX control kit v2, on a 75 bp paired-end indexed run. After sequencing, the resulting images were analysed with the proprietary Illumina pipeline v1.3. Reads for each of the indexed samples were then separated using a custom Perl script.

### Assembly

Reads from each indexed sample were trimmed to remove poor quality sequence at the 3' end. To determine the optimum trim length, initial de novo assemblies were made for read sets of different length (untrimmed reads, and reads trimmed to 70, 65, 55, 50 bp). These assemblies were carried out using Velvet 0.7 [18] with a range of hash lengths from 33 to 63 and a minimum k-mer coverage of 5×. For these initial assemblies, the data were treated as single reads, that is, the paired-end information was not used. Maximum contig lengths and N50 values were tabulated and the hash lengths that gave the highest N50 for each trimmed set of reads were selected for further optimisation. A second round of assembly was carried out on each trimmed set of reads using the hash length determined above and varying the coverage cut-off parameter from 1 to 100. Finally, paired-end assembly was carried out for each of these read-length/hash-length combinations using the coverage cut-off value that gave the highest N50 value. For these paired-end assemblies, expected coverage was set to the length-weighted median of the coverage values obtained in the initial single read assemblies, and the insert length was estimated as 240 bp. Assembled contigs were aligned to the *Cucumis sativus *chloroplast genome [GenBank: NC_007144; GenBank: DQ119058] using Geneious 4.7 [19].

**Figure 1:**
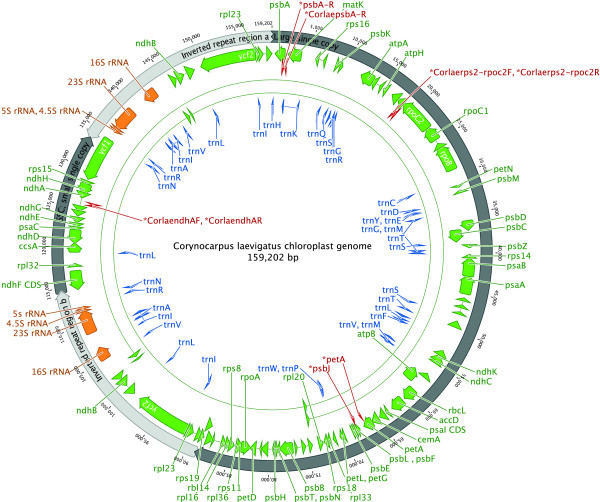
**Complete gene map of the entire *Corynocarpus laevigatus *chloroplast genome**. Arrowheads depict the direction of transcription. Binding sites for the primers used to generate the PCR products for resolving homopolymers are indicated in red. This figure was produced using Geneious [19].

Four short regions of ambiguous sequence were checked by PCR amplification using the following primers, custom designed using Primer3 [16] unless referenced: Corlaerps2-rpoc2F (TATAGGGTGCCATTCGAGGA), Corlaerps2-rpoc2R GTATCAACAACGGCCAATCC; CorlaendhAF (GGAATAGGATGGAGATAAGAAAGAC), CorlaendhAR (CACGATTCCGATCCAGAGTA); psbJ ATAGGTACTGTARCYGGTAT [20], petA AACARTTYGARAAGGTTCAATT [20]; psbAR (CGCGTCTCTCTAAAATTGCAGTCAT) [21], CorlaepsbA-R (ATCCGACTAGTTCCGGGTTC). Figure 1 shows the relative position of the priming sites on the karaka chloroplast genome. The PCR cycling conditions were modified slightly from an existing published protocol [20] as follows: template denaturation at 80°C for 5 min followed by 32 cycles of denaturation at 95°C for 1 min, primer annealing at 50°C for 1 min, followed by a ramp of 0.3°C/s to 65°C, and primer extension at 65°C for 4 min; followed by a final extension step of 5 min at 65°C. Amplified PCR products were sequenced using the BigDye Terminator Cycle Sequencing Kit (Applied Biosystems) and an ABI 3730 automated capillary sequencer at Massey Genome Service (Massey University, Palmerston North, New Zealand). The resulting sequences were visualised and edited using Sequencher 4.9 software for Mac (Gene Codes Corporation, Ann Arbor, MI). Using Geneious [19], the four ambiguous regions of the assembled genome were edited, where necessary, to match the Sanger sequences.

### Mapping and annotation

In order to check the de novo assembly, reads were aligned against the assembled genome using BWA [22] with default parameters. Only 19.6% of reads were successfully aligned, but this was sufficient to give a mean coverage of 400×. This mapping enabled us to resolve some short regions of ambiguous sequence in the assembly. The final complete chloroplast genome sequence was annotated using DOGMA [23] and through comparison to published complete chloroplast genome sequences available through GenBank [24].

## Results

### DNA sequencing template for karaka

**Figure 2:**
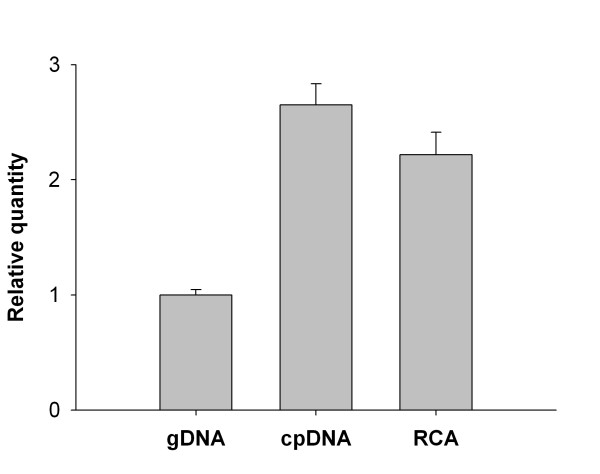
**Enrichment of chloroplast DNA from karaka**. The relative quantity of chloroplast DNA was determined in preparations of genomic DNA (gDNA), chloroplast-enriched DNA (cpDNA) and RCA amplified chloroplast-enriched cpDNA (RCA). Quantitative PCR amplification of the plastid encoded *psbB *gene was compared to amplification of nuclear 18S *rRNA*. The mean of four technical replicates ± SE has been reported as fold-differences relative to gDNA.

The relative quantity of chloroplast DNA in samples of total gDNA, cpDNA and RCAcpDNA preparations was determined by quantitative PCR (Figure 2). The enriched cpDNA sample had 2.6-fold higher levels of chloroplast DNA compared to a standard gDNA preparation prior to RCA and 2.2-fold after RCA. The purity of the DNA preparations was assessed by spectrophotometric A_260_/A_280 _and A_260_/A_230 _ratios. The gDNA and cpDNA samples had low ratios, indicating the presence of protein (A_260_/A_280 _= 1.66 and 1.69, respectively) and other contaminants such as carbohydrates and phenolics (A_260_/A_230 _= 1.49 and 1.28, respectively). RCA of the cpDNA-enriched sample substantially increased the quantity and quality of template DNA (A_260_/A_280 _= 1.75, A_260_/A_230 _= 2.20).

### Sequencing and assembly of the karaka chloroplast genomes

Paired-end sequencing of the RCAcpDNA template in a single lane on an Illumina GAII flow cell produced 1.84 and 1.76 million reads for the Chatham Islands sample and Kermadec Islands sample respectively. The Chatham Islands sample was assembled de novo as described in the methods section. The Kermadec Island sample was then mapped to this assembly.

The most useful assembly was achieved for the Chatham Islands sample, with reads trimmed to 50 bp, coverage cut-off of 9 and expected coverage of 40. While some of our other assemblies had higher overall N50 values or longer maximum contig lengths, these improved statistics did not reflect any real improvement in the assembly, as the large single-copy region was merged with part of the inverted repeat to form a single long contig at the expense of a more fragmented assembly of the remainder of the inverted repeat and small single-copy region.

The optimal assembly parameters produced a total of 13 contigs, four of which could be mapped to the *Cucumis sativus *chloroplast genome. These four contigs ranged from 7,857 bp to 88,955 bp in length and covered the entire chloroplast genome. The nine remaining contigs were much shorter, ranging from 81 bp to 669 bp. These were checked against the GenBank nucleotide database using the web-based BlastN algorithm [25], and the only significant alignments found were to nuclear ribosomal DNA sequences.

Of the four contigs mapping to the *C. sativus *chloroplast genome, one mapped to the inverted repeat region, one to the large single-copy region, and two to the small single-copy region. The overlaps between contigs at all four junctions between inverted repeat and single-copy regions were 40 bp long, indicating that contig extension was interrupted by the ambiguity of the overlap rather than by insufficient coverage. The overlap between the two contigs that formed the small single-copy region consisted of a polyA-polyT homopolymer. The contig corresponding to the large single-copy region contained six short (1-49 bp) stretches of ambiguous bases where Velvet was unable to resolve the sequence due to mono- or dinucleotide repeats. Three of these stretches were resolved by mapping the reads to the assembled sequence as described in the methods. The other three stretches, along with the overlap between the two contigs that made up the small single-copy region, were checked by PCR amplification and Sanger sequencing.

The final assembled chloroplast genome sequence (shown in Figure 1) was checked by mapping the original Illumina reads against the assembled sequence. A total of 344,475 of 1.76 million reads (19.6%) were successfully aligned, suggesting that approximately 80% of the DNA sequenced was of nuclear or mitochondrial origin.

## Discussion

We have shown that the modified chloroplast isolation protocol produced DNA template sufficiently enriched for chloroplast sequence to allow de novo assembly of the chloroplast genome. Comparison of the two genomes indicated high fidelity with less than 0.002% error. Whilst RCA of the cpDNA marginally reduced the final ratio of cpDNA/gDNA in the enriched sample, the purity of the DNA was of a higher quality for Illumina sequencing.

The coverage cut-off parameter of the Velvet assembler was crucial for successful assembly, as it allowed the chloroplast sequence reads to be assembled without interference from nuclear sequence. Although over 80% of reads failed to align to our assembled chloroplast genome, and are likely to be of nuclear origin, the much greater size of the nuclear genome means that these reads were present at much lower coverage than the chloroplast reads. A notable exception is nuclear ribosomal DNA, which is present in many copies in the nuclear genome, thus its coverage was comparable to that of the chloroplast genome in our enriched sample.

The lower copy number of the nuclear genome compared to the chloroplast genomes means that nuclear copies of chloroplast DNA sequences are very unlikely to affect our assemblies. In contrast, nuclear-encoded chloroplast DNA may be more difficult to distinguish from chloroplast-encoded sequences if amplified by chloroplast DNA primers. Thus, this is potentially another advantage of the approach we have used for determining complete chloroplast genome sequences.

Finally, although de novo assembly was a feature of our protocol, the availability of a related reference genome did help with our final assembly, allowing us to separate contigs derived from chloroplast DNA from the few short contigs of nuclear origin. This was helpful for determining the arrangement of chloroplast contigs.

## Conclusions

We have successfully applied a whole genome sequencing approach to determine the complete chloroplast genome sequence of karaka. We have also applied this approach more recently to a range of New Zealand seed plants (gymnosperms and angiosperms: herbaceous and woody plants), sequencing up to three chloroplast genomes per GAII flow cell lane. Thus we are confident that the approach that we describe here for karaka provides a fast and efficient protocol for obtaining whole chloroplast genome sequences for seed plants.

The fully annotated chloroplast genome sequence of karaka (*Corynocarpus laevigatus*) from the Chatham Islands sample has been deposited in the GenBank database under accession number HQ207704.

## Competing interests

The authors declare that they have no competing interests.

## Authors' contributions

RAA helped with the design of the study, isolated chloroplasts, extracted the DNA, performed RCA amplification of cpDNA, carried out PCR experiments and submitted data to GenBank, as well as drafting, contributing to and editing the manuscript; BJM carried out de novo assembly and annotation of the genome, contributed to and edited the manuscript; LDS contributed to, edited and revised several versions of the manuscript and provided academic guidance to RAA during her PhD study. LB carried out sample preparation and sequencing on the Illumina GAII; NWA designed and performed qPCR assays and contributed to and edited the manuscript; PJL conceived of and designed the study, contributed to, edited and revised several versions of the manuscript and provided academic guidance to RAA during her PhD study. All authors read and approved the final manuscript.
